# Rapid identification of mosquito species and age by mass spectrometric analysis

**DOI:** 10.1186/s12915-022-01508-8

**Published:** 2023-01-24

**Authors:** Iris Wagner, Linda Grigoraki, Peter Enevoldson, Michael Clarkson, Sam Jones, Jane L. Hurst, Robert J. Beynon, Hilary Ranson

**Affiliations:** 1 Centre for Proteome Research, Institute of Systems, Molecular and Integrative Biology, University of Liverpool, Liverpool, L69 7ZB UK; 2 Department of Vector Biology, Liverpool School of Tropical Medicine, Pembroke Place, Liverpool, L3 5QA UK; 3 Walton Centre NHS Foundation Trust, Lower Lane, Liverpool, L9 7LJ UK; 4 Department of Livestock and One Health, University of Liverpool, Institute of Infection, Veterinary and Ecological Sciences, Leahurst Campus, Neston, CH64 7TE UK; 5 International Pheromone Systems Ltd, Evolution House, Long Acres Road, Clayhill Industrial Estate, Neston, CH64 3RL Cheshire UK; 6 Mammalian Behaviour and Evolution Group, Institute of Infection, Veterinary and Ecological Sciences, University of Liverpool, Leahurst Campus, Neston, CH64 7TE UK

**Keywords:** Rapid evaporative ionisation mass spectrometry, REIMS, Mass spectrometry, Species identification, Mosquito, Age grading

## Abstract

**Background:**

A rapid, accurate method to identify and to age-grade mosquito populations would be a major advance in predicting the risk of pathogen transmission and evaluating the public health impact of vector control interventions. Whilst other spectrometric or transcriptomic methods show promise, current approaches rely on challenging morphological techniques or simple binary classifications that cannot identify the subset of the population old enough to be infectious. In this study, the ability of rapid evaporative ionisation mass spectrometry (REIMS) to identify the species and age of mosquitoes reared in the laboratory and derived from the wild was investigated.

**Results:**

The accuracy of REIMS in identifying morphologically identical species of the *Anopheles gambiae* complex exceeded 97% using principal component/linear discriminant analysis (PC-LDA) and 84% based on random forest analysis. Age separation into 3 different age categories (1 day, 5–6 days, 14–15 days) was achieved with 99% (PC-LDA) and 91% (random forest) accuracy. When tested on wild mosquitoes from the UK, REIMS data could determine the species and age of the specimens with accuracies of 91 and 90% respectively.

**Conclusions:**

The accuracy of REIMS to resolve the species and age of *Anopheles* mosquitoes is comparable to that achieved by infrared spectroscopy approaches. The processing time and ease of use represent significant advantages over current, dissection-based methods. Importantly, the accuracy was maintained when using wild mosquitoes reared under differing environmental conditions, and when mosquitoes were stored frozen or desiccated. This high throughput approach thus has potential to conduct rapid, real-time monitoring of vector populations, providing entomological evidence of the impact of alternative interventions.

**Supplementary Information:**

The online version contains supplementary material available at 10.1186/s12915-022-01508-8.

## Background

Mosquito-borne diseases cause suffering and hundreds of thousands of deaths every year. Malaria remains a leading cause of mortality and morbidity in Africa, killing over 600,000 people annually [1] and arboviral diseases transmitted by *Aedes* mosquitoes, like dengue, chikungunya and Zika have placed more than half of the world’s population at risk [2]. Vector control and in particular the use of insecticides in domestic environments (insecticide-treated bed nets and indoor residual spraying) and mosquito breeding sites, remains the most effective tool in averting mosquito-borne infections [3]. However, increasing insecticide resistance [4, 5] is intensifying the search for new vector control tools [6, 7] and it becomes critical to have methods for evaluating the efficacy of mosquito control interventions. Epidemiological studies are considered the gold standard in evaluating the impact of vector control on disease transmission, but they are logistically and financially challenging. An alternative faster and cheaper approach could be the collection of robust entomological data that can directly reflect the risk of disease transmission.

Mosquito survival is a major determinant of their vectorial capacity. This is because after the ingestion of an infected blood meal the pathogens undergo a complex pathway of replication and dissemination to the salivary glands from where they can be transmitted further. The length of time that elapses between a mosquito ingesting a pathogen and becoming infectious is known as the extrinsic incubation period (EIP). For the malaria-causing *Plasmodium* parasites, the EIP is at least 10 days [8] whereas for viruses it ranges from 6 to 15 days [9, 10]. Thus, data on the age profile of a mosquito population would provide a much clearer indication of the risks of transmission, and the impact of interventions in reducing these risks, than simple counts of adult mosquito density. Indeed, the limited data available on the daily survival rates of mosquitoes under natural conditions suggest that only a very small proportion survives beyond the EIP of most human pathogens [11–13].

Traditionally mosquito age has been estimated based on morphological changes observed in the ovaries of female mosquitoes. These changes can be used to assess whether a mosquito has laid eggs (parous); skilled technicians are also able to determine the number of gonotrophic cycles from examination of the ovaries. This age grading method avoids the need for specialised equipment and can be done with minimal cost under field settings, but it is labour-intensive and only provides an indirect measure of the mosquitoes’ physiological age. A range of additional methods have been explored for their potential use in mosquito age grading, including cuticular hydrocarbon analysis [14], gene expression [15–17] and protein profiling [18–20], MALDI-TOF mass spectrometry [18, 21–23], near-infrared (NIRS) and mid-infrared (MIRS) spectroscopy (recently reviewed in [24]) and quantification of spermatazoa [25]. NIRS and MIRS show the most promise, mainly due to the fast data acquisition they provide [26–28]. NIRS has a high predictive power when assigning mosquitoes into binary age categories (> 7 and < 7 days old) [29] and challenges have been encountered when factoring in environmental variability [30]. In earlier evaluations MIRS could clearly differentiate very old (> 15 days) and very young (1 day) mosquitoes, but had less accuracy in distinguishing intermediate ages [26]; however recently, a deep learning MIRS model has been developed that yields greater granularity to age grading in field-caught mosquitoes [28].

Many mosquitoes belong to groups of cryptic species and, in some cases, only some members of these groups are disease vectors. For example, *Anopheles gambiae *sensu lato complex comprises at least seven sibling species of mosquitoes that are morphologically indistinguishable. Only three of these species (*An. gambiae s.s*., *An. coluzzii* and *An. arabiensis*) are important malaria vectors; each of these has differing ecology, behaviour, host-feeding preference and tolerance to insecticides [31]. Therefore, changes in species composition can have important implications for the selection of the most appropriate control tools. Multiple PCR assays have been developed to differentiate members of the *An. gambiae* species complex, but these can be challenging to perform at scale.

A method that could provide a rapid assessment of both the species and the age of mosquitoes, that is robust to changes in the environment to which they have been exposed and insensitive to storage conditions post capture would greatly accelerate the evaluation of mosquito control strategies. In this study, the potential of rapid evaporative ionisation mass spectrometry (REIMS) to predict the age and species of mosquitoes was evaluated. REIMS is based on the combustion of the sample using diathermy, ionisation and subsequent mass spectrometry of the ions that are generated. It is extremely rapid (5 s per sample), generates richly detailed mass spectra and has been used in such diverse areas as surgery (where it was first developed as the iKnife — [32–34]), food fraud [35–37], in bacterial typing [38–40], monitoring of heterologous protein expression by bacteria [41] and in the analysis of rodent faecal material [42]. Most recently, we have completed a proof-of-concept series of experiments with laboratory-reared *Drosophila *spp. to demonstrate that REIMS can be used to discriminate species and sex in insects without any prior processing [43].

In this study, the ability of REIMS to provide information relevant to mosquitoes was explored. Initially, laboratory-reared mosquitoes from the *An. gambiae* complex were tested as proof of principle under controlled conditions. To test the applicability of the method for field studies, REIMS was used to analyse multiple species of mosquitoes collected in North West England. We demonstrate the potential of REIMS for the classification of species and adult age, traits of key importance in profiling mosquito populations.

## Results

### REIMS spectra are a rich and informative data source

**Fig. 1:**
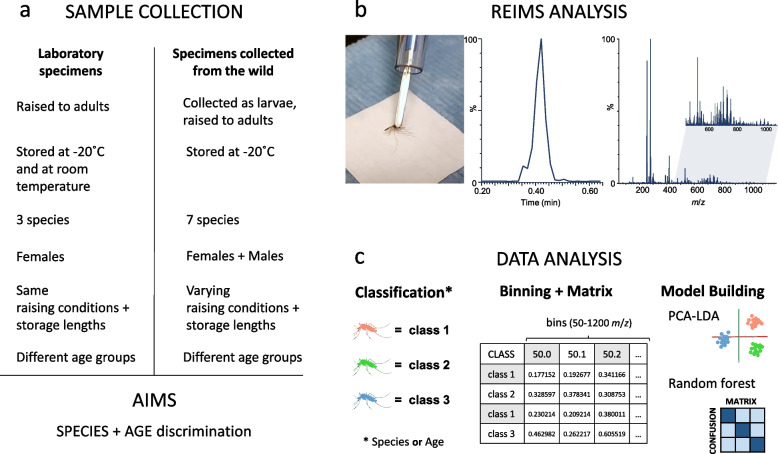
Overview of REIMS analysis of mosquito specimens. (**a**) General information about the mosquito samples used in this study. Details are listed in the methods section or mentioned with the corresponding results. Collection and treatment procedures were varied to explore the stability of the REIMS analysis. (**b**) Samples were combusted by the diathermy electric current and the resulting aerosol was evacuated through a tube and introduced to the mass spectrometer, where the ionised molecules were detected. One ‘burn event’ was generated for each mosquito and the corresponding mass spectrum was integrated over the duration of the event (typically, 5 s). (**c**) Mass spectral data were pre-treated and collapsed into 0.1 m*/z* wide bins from 50 m*/z* to 1200 m*/z*. The resulting data matrix is then used to identify patterns through principal component and linear discriminant analysis (PCA-LDA) as well as random forest analysis, allowing sample classification and identification

To generate a REIMS signal, the insect sample is combusted by the application of a high-frequency electric current. The resulting aerosol is collected by an airflow leading to the ion source of the instrument, where molecules are de-clustered/ionised and subsequently detected by the mass spectrometer [43]. In a single burn event, a small insect, such as an adult *An. gambiae* (adult dry weight, 250 µg, [44]) can be completely consumed in about 5–10 s (Additional file 1: video file V1). The mass spectra acquired at 1 Hz throughout the burn event are summed, processed and binned into 0.1 mass to charge (*m*/*z)* bins. The negative ion mass spectra largely reflect the lipid profile of the insect and are richly informative in the range of 50–1200 m*/z*. Moreover, different individuals from the same cohort yielded consistent mass spectra, but which also differed from the mass spectral patterns from other, different cohorts. The rapidity of data collection means several hundred individuals can be analysed per day. After data acquisition, the resultant data matrix, containing sample identification and binned *m*/*z* values (typically over 100 samples and over 11,000 m*/z* bins) is then subjected to multivariate analysis using machine learning and classification approaches (Fig. 1).

### Application of REIMS for species identification

#### Laboratory-reared mosquitoes

First, we examined whether REIMS was able to discriminate three species of the *An. gambiae* species complex. We acquired REIMS mass spectra from 202 specimens, all 4-day-old females, comprising 54 specimens of *An. coluzzii* (strain Ngousso), 59 specimens of *An. gambiae s.s* (strain Kisumu) and 89 *An. arabiensis* mosquitoes, (strain Moz). All mosquitoes were killed by freezing, stored at − 20 °C and analysed by REIMS in a randomised order. In all instances, the burn yielded a satisfactory mass spectrum. Prior to data analysis, mass spectral data were pre-processed in Offline Model Builder (Waters) in which the background signal was subtracted, spectra were mass corrected using a lock-mass standard (leucine enkephalin, 554.26 m/*z*) that was co-infused with each sample and finally, spectra were discretised by binning signals into 0.1 m/*z* wide intervals.

**Fig. 2:**
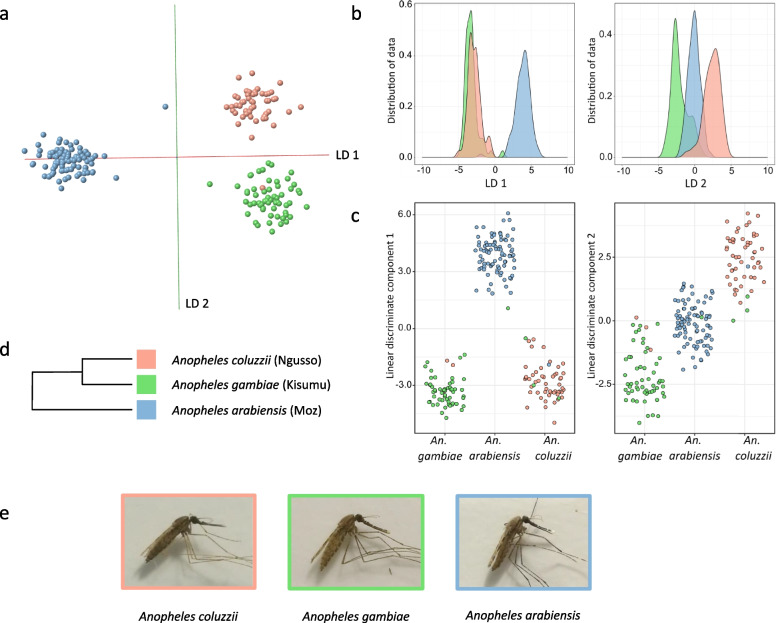
Analysis of three *Anopheles* mosquito species by REIMS. A total of 202 specimens from *An. arabiensis* (*n* = 89), *An. coluzzii* (*n* = 54) and *An. gambiae s.s* (*n* = 59) were killed through freezing and stored at − 20 °C until REIMS analysis. All specimens were female and 4 days old. Principal component–linear discriminant (PC-LD) analysis of the REIMS data within the model building software Offline Model Builder led to a clear separation of the three species (**a**). After exporting the data matrix (incl. classifications and signal intensities after pre-processing) PC-LDA was repeated in R; results are displayed in form of kernel density (**b**) and scatter plots (**c**), shown for both linear discriminants 1 and 2. The group formation correlates with the genetic relatedness of the three groups (**d**, from [45]); the biggest variance (LD 1) supporting the separation of *An. arabiensis*, followed by the separation of *An. gambiae s.s* and *An. coluzzii* via LD 2. Images of females from all three species are displayed in (**e**)

Principal component analysis followed by linear discriminant analysis (PC-LDA) using the Offline Model Builder software resolved the three species with only a single *An. coluzzii* individual being co-localised with the individuals from *An. gambiae s.s* (Fig. 2a). The first discriminant function was responsible for resolution of *An. arabiensis* from the other two *An. gambiae s.l* species whereas the second function yielded a good resolution of *An. gambiae s.s* and *An. coluzzii*, a result which correlates with their genetic relatedness (Fig. 2d). This finding is mirrored in the kernel density (Fig. 2b) and scatter plots (Fig. 2c) based on PC-LDA. The mass spectra for the three species were very similar and exhibited slight variation in the region of 600–1200 m*/z* (the averaged spectra based on all individuals available for each species are in Additional file 2: Sup. Figure 1). The subtlety of REIMS discrimination and the ability to resolve different species, even when they are closely related and morphologically identical (Fig. 2d, e), resonates well with our prior observations on *Drosophila* species [43]. As a further test, the entire set of REIMS spectra from all individuals were randomly assigned to three equally sized categories — these could not be resolved (Additional file 2: Sup. Figure 2). The PCA-LDA model was also re-built using less variance (fewer principal components) and all models were cross-validated in Offline Model Builder (Additional file 2: Sup. Figures 3 and 4).

#### Wild mosquito populations

Whilst laboratory specimens are reared under controlled conditions and feeding regimens, a critical test of the methodology arises when applied to specimens recovered from the natural environment. We therefore applied REIMS to the analysis of mosquitoes derived from the saltwater marshes and freshwater pools around the town of Neston on the Wirral peninsula (located in the Northwest of the U.K). This area and terrain support the proliferation of several different mosquito species that have been monitored over many years [46]. Larvae were collected and raised to adults of both sexes, which were identified to species level by morphological examination [47, 48]. In total, seven different species were collected in sufficiently large numbers for REIMS analysis: *Culex pipiens*,* Culiseta annulata*,* Aedes caspius*,* Aedes punctor*,* Aedes rusticus*,* Aedes cantans* and* Aedes detritus.* Eighty individuals of each species were analysed in a formally randomised sequence.

**Fig. 3:**
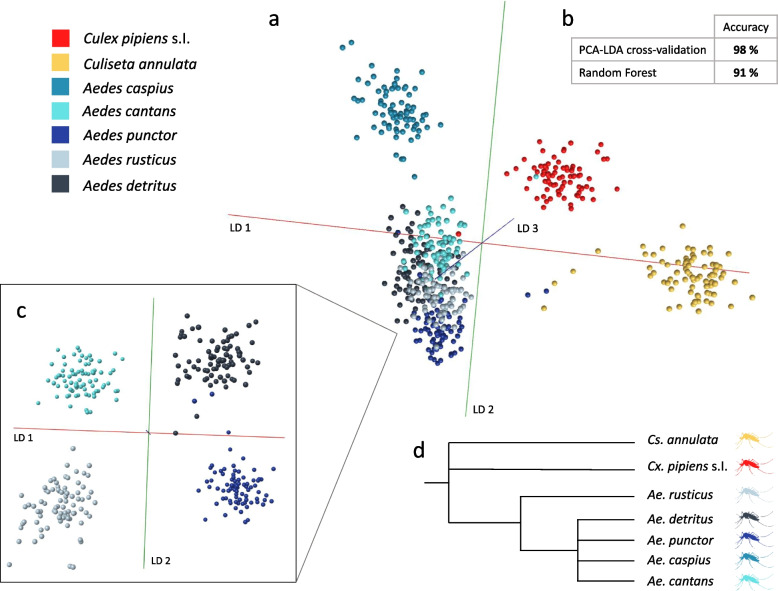
Resolution of local mosquito species using wild-derived specimens. Mosquito larvae collected from the wild (Neston region, north west UK) were raised to adults (males and females, ages from 0 to 4 days) and identified by morphological examination, before being killed and stored at − 20 °C for varying lengths of time. Collection of larvae as well as REIMS analysis of stored adults occurred over several months. The data acquired for a total of seven species (80 individuals per species) was analysed in Offline Model Builder via PC-LDA using 100 PCs (**a**, **b**). Visualisation was aided by removing clearly separated species groups (*Cs. annulata*, *Cx. pipiens*, *Ae. caspius*) from the model (**c**) and axis rotation. The PC-LDA separation was resonant with the phylogenetic relationships of these species (**d**). The OMB model was cross-validated (using the option ‘Leave 20% out’ and a standard deviation of 5) and reached a classification accuracy of 98%. Additionally, the data was analysed using a random forest algorithm, which produced a separation accuracy of 91% (section b). Details of the cross-validation and random forest results can be found in Additional file 2: Sup. Figure 5

The data, acquired from 0- to 4-day-old female and male mosquitoes belonging to seven different species (80 individuals per species) were used to develop a species model (Fig. 3). PC-LDA in Offline Model Builder (OMB) readily resolves *Cx. pipiens*, *Cs. annulata* and *Ae. caspius* from a tight cluster containing the other four species (Fig. 3a). In fact, this four-species cluster was also resolved, as seen with a clipped and differentially projected location (Fig. 3c). Indeed, tighter clustering of data points for *Ae. cantans*,* Ae. punctor*,* Ae.rusticus *and* Ae. detritus* is consistent with their phylogenetic proximity [49] (Fig. 3d). The samples were also analysed through random forests with the resulting species determination model reaching an average accuracy of 91% compared to 98% for OMB (Fig. 3b, Additional file 2: Sup. Figure 5). When species classifications were randomly assigned to samples, separation of classes failed (Additional file 2: Sup. Figure 6), confirming that there are discriminant patterns within spectra from mosquitoes displaying natural variability, and establishing the potential of REIMS to the study of field-caught mosquitoes.

### Blinded identification study

To test the utility of REIMS as a predictive tool, we used the seven-species model in Fig. 3. A further 187 larvae were grown to adults and assigned to species by independent morphological examination. Species was blinded to the analyst, and the REIMS data were submitted to the model for species prediction. Overall, 94% accuracy was achieved for blinded samples (Table 1).

**Table 1:**
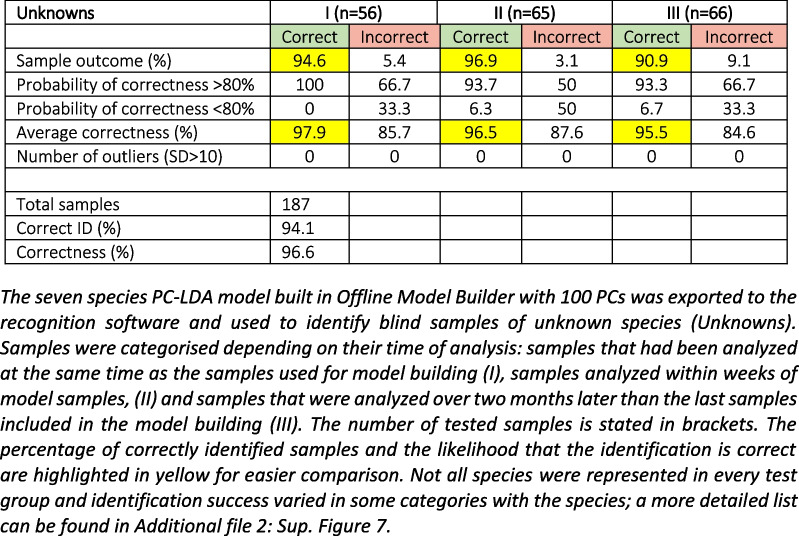
Species identification of unknown samples

The seven-species PC-LDA model built in Offline Model Builder with 100 PCs was exported to the recognition software and used to identify blind samples of unknown species (Unknowns). Samples were categorised depending on their time of analysis: samples that had been analysed at the same time as the samples used for model building (I), samples analysed within weeks of model samples, (II) and samples that were analysed over two months later than the last samples included in the model building (III). The number of tested samples is stated in brackets. The percentage of correctly identified samples and the likelihood that the identification is correct are highlighted in yellow for easier comparison. Not all species were represented in every test group and identification success varied in some categories with the species; a more detailed list can be found in Additional file 2: Sup. Figure 7.

### Species identification of larval stage mosquitoes

We explored the ability of REIMS to resolve the immature (larval) stages of mosquitoes into species. Whilst it is very difficult to establish the species of the larvae by morphology, many of the pools in the Neston area from which the mosquitoes were collected are essentially mosquito monocultures. Many larvae were harvested, a subset of which was immediately analysed with REIMS, whilst the remainder were allowed to develop into adults. The identity of the emerged adults was then used to predict the species heterogeneity of the larval population in each marsh pool, confirming that the pools contained single species (100% of the adults identified were the same species, either *Ae. puncto*r or *Ae. detritus;* detailed data in Additional file 2: Sup. Figure 8).

**Fig. 4:**
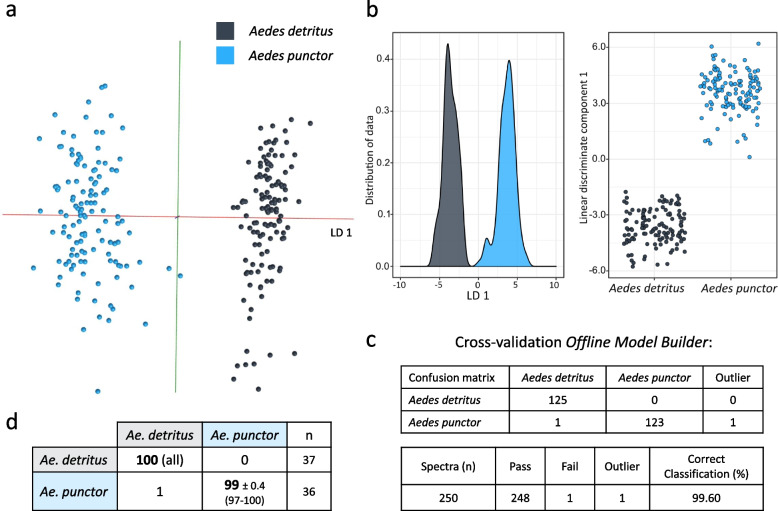
Species identification of immature mosquito larvae. Immature mosquitoes were obtained by filtering water collected from different pools, followed by 2–3 rinsing steps before killing and storing them at − 20 °C. Samples, mostly 3rd instar larvae were analysed with the same REIMS settings used for adult specimens. Their species was confirmed by sampling the same pool of larvae and raising them to adults before, during and after taking samples to be used for this model. The differences detected by PC-LDA are visualised in the form of an OMB model (**a**) and kernel density and scatterplots produced in R (**b**). The separation was put to test via cross-validation in OMB (**c**) and random forest analysis with training/test dataset split of 70%/30% (**d**). The separation of *Ae. detritus* (*n* = 125) and *Ae. punctor* (*n* = 125) larvae required little information/variance, reaching very distinct separation with only 20 principal components for PC-LDA. When testing the random forest model, samples were identified correctly most of the time leading to correct identification rates of 100% (*Ae. detritus*) and 99% (*Ae. punctor*)

Separation of *Ae. detritus* and *Ae. punctor* larvae was very distinct with only 20 principal components (Fig. 4a, b). In random forest analysis, the test samples were virtually always identified correctly, with correct identification rates of 100% (*Ae. detritus*) and 99% (*Ae. punctor*) (Fig. 4d). Cross-validation of the OMB model resulted in one misclassification and one outlier from 250 samples (Fig. 4c). Seeing this distinct separation through PC-LDA, principal component analysis alone was performed in OMB, which resulted in samples clustering into species groups along PC3 (Additional file 2: Sup. Figure 8b). The larval-based species model was also re-built using randomly assigned classifications; the resulting model was devoid of any separation or sample clustering (Additional file 2: Sup. Figure 8c).

### Application of REIMS for age profiling

A characteristic of particular significance in vector control is that of female mosquito age. Establishing the age of individual mosquito specimens could, in combination with regular sampling and high-throughput analysis, allow determination of population age profiles and hence the risk of disease transmission. Age distribution would inform and support vector control strategies, particularly when in control validation stages, with a reduction in the median age of the population being a more reliable proxy of the public health value of a new tool than metrics based on population density. Indeed, vector control trials frequently include measurements of the proportion of the population that are parous (having laid at least one egg batch) as a crude measure of the proportion of ‘older’ adults (over 3 days [50]). The ability to rapidly and reliably separate mosquitoes into different age groups would be transformative for mosquito surveillance. We therefore explored whether REIMS possessed the ability to resolve mosquitoes according to age.

#### Laboratory-reared specimens

**Fig. 5:**
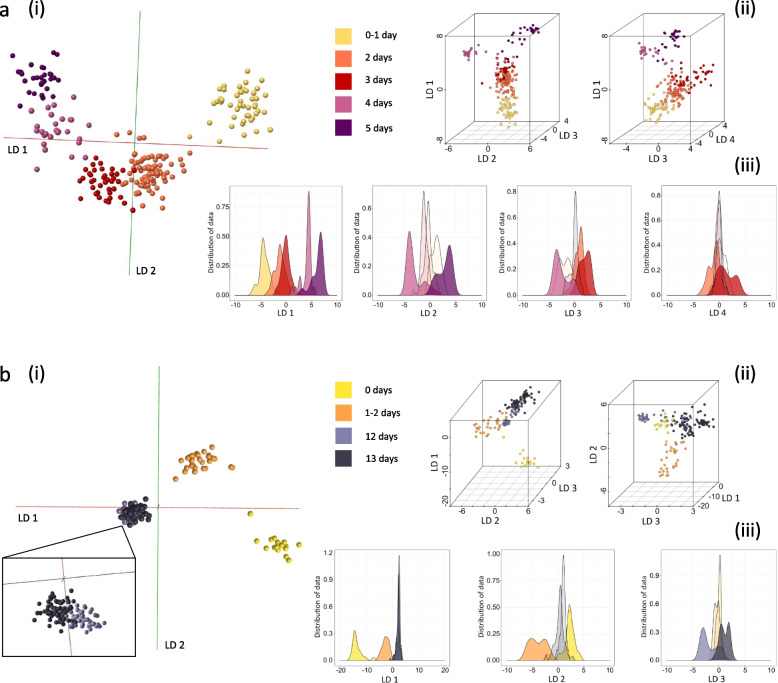
Discrimination of *Anopheles gambiae* mosquitoes by age. Two groups (**a** and **b**) of *Anopheles gambiae s.s* (Kisumu strain) specimens of different ages were killed by freezing before REIMS analysis. Mosquitoes were raised on sugar solution, regardless of age. Differences between age groups were explored by PC-LDA and visualised using OMB (i) as well as R, in form of 3D models (using different linear discriminant combinations) (ii) and kernel density plots for each LD. The difference between classes in model **a** (built with 100 PCs in OMB and R) is only 24 h; nonetheless, a distinct group formation can be observed with chronological positioning within the 3D space, leading to a transition from younger to older samples. Model **b** (built with 88 PCs in OMB and 85 PCs in R), comprising young and old mosquitoes (2 groups each), revealed greater variance between the young classes than between the old. Sample numbers per class, model **a**: 0–1 day (*n* = 47), 2 days (*n* = 84), 3 days (*n* = 39), 4 days (*n* = 27), 5 days (*n* = 30); model: 0 days (*n* = 17), 1–2 days (*n* = 29), 12 days (*n* = 46), 13 days (*n* = 83)

Reports of the lifespan of female *An. gambiae s.l* mosquitoes range from 2 to 43 days, with longevity under laboratory conditions typically greatly exceeding that in the field where predation, environment and disease all reduce survival [51]. For the initial tests, we included female *An. gambiae s.s* (Kisumu strain) raised for 0–1 day, 2 days, 3 days, 4 days and 5 days under standard insectary conditions. Mosquitoes of all five age groups were killed by freezing and stored at − 20 °C the same day. After REIMS analysis, PC-LDA in OMB led to a clear clustering according to age, with the location of each age group reflecting the developmental age of specimens (Fig. 5a). Further PC-LDA in R and visualisation through 3D plots with different triads of the top four linear discriminants reveals a similar picture of age progression along LD 1. The values of LD 1 contained enough information to provide some distinction for all groups but with overlap; the second to fourth LDs added further resolution of specific groups, improving the overall separation.

To test the discrimination with greater age separation, a second set of *An. gambiae s.s* was analysed, focusing on more broadly spaced age groups: very young (0 days or 1–2 days) and old (12 days or 13 days) (Fig. 5b). We adopted different age classes to protect against an artefactual separation across a single age class. Again, samples were grouped according to their age class, however, with unequal separation between the three classes. The difference between mosquitoes that had just emerged (day 0) and those that are 1–2 days old was greater than the difference between adults at 12 days and 13 days, which could reflect metabolic and developmental changes in the first 24 h after emergence from pupae. This difference in variance can also be clearly seen in either scatter or kernel density plots; both LD 1 and 2 are adding to the separation of the young mosquitoes, whereas LD 3 was able to provide limited variance to distinguish between 12- and 13-day mosquitoes.

Both age models were also built with fewer principal components (Additional file 2: Sup. Figure 9), to test robustness. Furthermore, as a control, mosquitoes were randomly assigned to the different age classes (Additional file 2: Sup. Figure 10) to confirm that separation is driven by age-related differences between classes.

The ease with which age could be resolved and the clear separation of the 0–1-day-old mosquitoes might reflect a major change in lipid deposits post-emergence. However, averaged mass spectra of 0- to 5-day-old mosquitoes are visually similar (Additional file 2: Sup. Figure 11). Nevertheless, sample clusters based on age-related variance were formed, making age a REIMS-accessible parameter. In contrast to species or sex, age is a continuous parameter and could benefit from conversion to discontinuous categorisation to help define class boundaries and increase separation and identification accuracy. A reduction in class numbers (from five to three and from four to three), achieved by combining neighbouring classes, reduced the overlap between age groups and improved the definition of class boundaries (Additional file 2: Sup. Figure 12) as well as increased correct classifications rates obtained through cross-validation of PC-LDA models in OMB (Additional file 2: Sup. Figure 13).

**Fig. 6:**
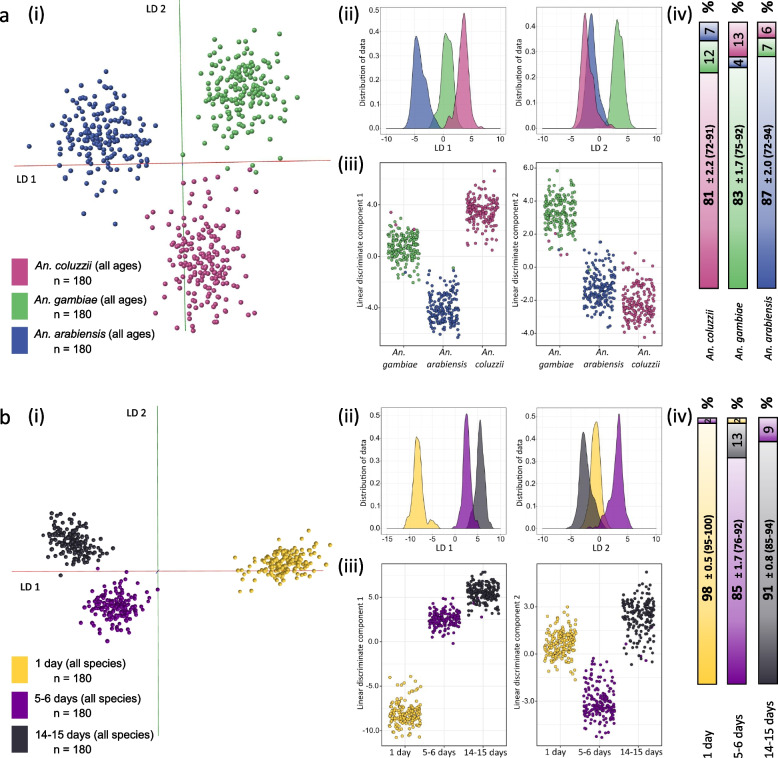
Separation of species and age by REIMS. Mosquitoes (total *n* = 540) were raised to three different age groups (1 day, 5–6 days, 14–15 days) for each of the three species (*An. coluzzii*, *An. gambiae s.s*, *An. arabiensis*, see text). The specimens were killed by dehydration and stored at room temperature with desiccant for 1–1.5 weeks prior to analysis. The samples were used to build two models: one separating the three species (**a**) and one separating the three age groups (**b**). Data was processed using PC-LDA in Offline Model Builder (using 100 PCs) (i) and R (using 235 PCs), latter visualised for each linear discriminant (LD 1 and LD 2) separately in form of kernel density (ii) and scatter plots (iii). The data matrix, exported from the Offline Model Builder software, was additionally analysed using the random forest algorithm in R; 70% of the samples were used for model building, 30% as test samples. The classification results are depicted as a bar graph, showing percentages of correctly and wrongly identified samples (iv). Depicted are the average values of 10 random forest repeat runs ± the standard error of the mean, with the range of accuracy values that were achieved in brackets

Following the successful separation of laboratory-reared mosquitoes according to their species or age, potentially confounding factors were introduced to the next sample set to further test the concept of mosquito characterisation through REIMS, particularly in the context of field-caught samples, in which circumstance, freezing might not be an option. Female specimens from *An. arabiensis (*strain Moz), *An. gambiae* s.s (strain Kisumu) and *An. coluzzii* (strain Ngousso) were each raised and sampled into three different age groups: 1 day, 5–6 days and 14–15 days. The specimens were then killed through dehydration and stored at room temperature with desiccant material for 1–1.5 weeks before REIMS analysis. These data were used to build two different models: one to resolve species, the other to explore resolution according to age. Thus, mosquitoes of different ages are part of the species model (Fig. 6a) and age separation was tested on aggregated data from all three species (Fig. 6b).

The average performance statistics and number of correct and incorrect classifications confirm a high level of discrimination (Fig. 6). Despite increasing variability in the data set by inclusion of specimens of different ages, separation of species was still successful, even though samples within groups are slightly more scattered and overall group resolution was slightly reduced. The average accuracy achieved through random forest analysis was 87% correct identification for *An. arabiensis*, 81% for *An. coluzzii* and 83% for *An. gambiae s.s*. The greatest degree of misclassification was clearly between *An. gambiae s.s* and *An. coluzzii* (12–13%). Cross-validation of the PC-LDA model built in OMB resulted in an even higher correct classification rate of 98% (Additional file 2: Sup. Figure 14).

The second model, separating mosquitoes according to their age, displays a tight clustering of samples within age groups and a very distinct separation between age groups. As previously observed, the very young mosquitoes (1 day old) resolved readily from older specimens and show the biggest separation. Both analytical approaches, PC-LDA and random forest, reinforce the earlier observation that the 1-day-old specimens are easily resolved (Fig. 6b). Overall, the age model achieved an average accuracy of 91%, making this age separation species independent. Cross-validation of the PC-LDA-based model produced an average accuracy of 99% (Additional file 2: Sup. Figure 14). Thus, although samples had been treated very differently, compared to the previous species and age models (Figs. 2 and 5), REIMS analysis nevertheless resulted in information-rich mass spectral data allowing for clear separation of species and highly accurate age discrimination. Both models (species and age) were also re-built in R with fewer principal components to confirm that classes can still be separated using less variance and potentially a smaller number of separating factors (Additional file 2: Sup. Figure 15).

**Fig. 7:**
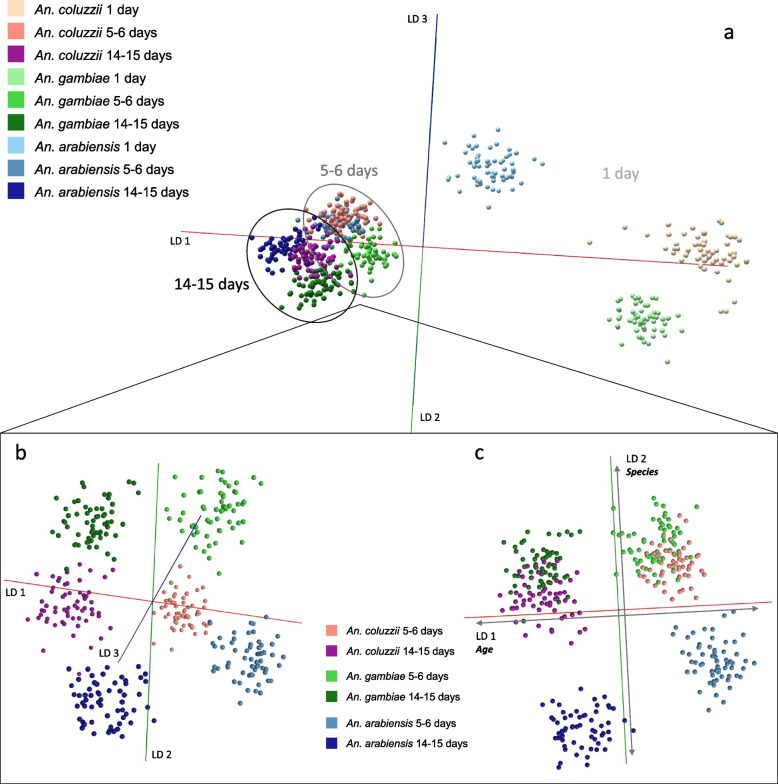
Two-factor model combining species and age information. The same samples used to build the species and age models in Fig. 6 were used to construct a two-factor model, comprising nine classes, each containing species and age information. Separation of all nine classes (60 specimens each) was attempted using PC-LDA in Offline Model Builder (based on 100 PCs) (**a**). Due to the wide dispersion of the 1-day-old groups, the spatial resolution of the 5–6- and 14–15-day-old groups in the 3D space is hindered. To help visualise the separation, the 1-day-old sample groups were removed (**b**). The largest variance in the data set (LD 1) is correlated with age, followed by species separation of *An. arabiensis* enabled by LD 2 and lastly separation of *An. gambiae s.s* from *An. coluzzii* based on LD 3 (**c**)

For species resolution, the *m/z* bins contributing a high level of discrimination were different from those driving age discrimination (Additional file 2: Sup. Figure 16), suggesting that it should be possible to derive a two-dimensional model. Both characteristics, species and age, would be of interest when identifying trapped mosquitoes, leaving two options: acquire data and test with two separate models or build a two-factor model capable of providing both types of information at once. To test whether a two-factor model could be a viable option, the same data set used to build the species and age models (Fig. 6) was split into nine classes, each representing two factors, species and age (Fig. 7). Regardless of the added difficulty of splitting variance to enable separation of nine classes based on two factors, samples were successfully grouped and separated. The average random forest performance of the species model was 84%, lower than the performance of the age model (91%). The two-factor model assigned more variance to the age separation than the separation of species, which is apparent in the clustering according to age rather than species, followed by the separation of the three clusters (1 day, 5–6 days, 14–15 days) along linear discriminant 1 (Fig. 7a). From comparison of the 5–6-day-old and the 14–15-day-old classes, age is separated based on LD 1, followed by species resolution of *An. arabiensis* from the other two *Anopheles* species through LD 2 and further separation of *An. gambiae s.s* from *An. coluzzii* via LD 3 (Fig. 7b, c). This analysis augurs well for profiling analyses with wild-caught specimens. Cross-validation following PC-LDA in OMB produced an average correct classification rate of 97% (Additional file 2: Sup. Figure 17) whereas random forest analysis identified 79% of samples correctly (Additional file 2: Sup. Figure 18, PC-LDA in Sup. Figure 19).

Following random forest analysis, the variables driving the separation of the nine classes were aggregated and compared to *m/z* bins previously identified as important in the species and age models (Additional file 2: Sup. Figure 16). Three out of five variables had been identified previously, which confirms their importance for species and age separation respectively (Additional file 2: Sup. Figure 20). Lastly, the separate age and species models as well as the nine-class model were all rebuilt using randomly assigned classifications to ensure the separating patterns are not based on unrelated noise; separation failed for all three models (Additional file 2: Sup. Figure 21).

#### Wild-derived specimens

**Fig. 8:**
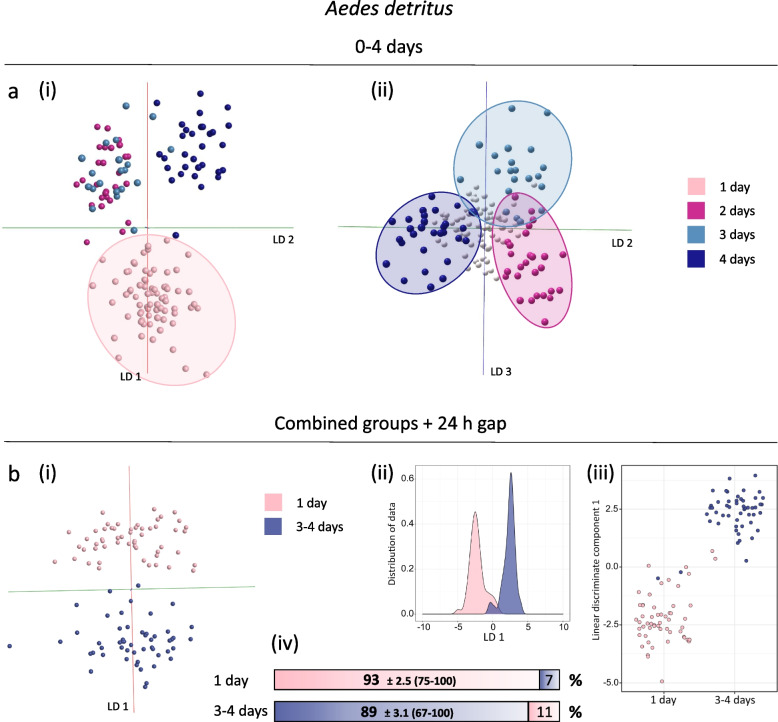
Discrimination of *Aedes detritus* mosquitoes by age. *Ae. detritus* mosquitoes, emerged from larvae collected from natural pools, were killed by freezing at different ages ranging from 0 to 4 days (males and females). As a first step samples were sorted into four age groups for PC-LDA (based on 75 PCs), which resulted in a definitive grouping according to age (**a**), with linear discriminant 1 separating just emerged mosquitoes (1 day) (i) and linear discriminants 2 and 3 separating specimens which are between 2 and 4 days (ii). To improve separation a 1-day gap was introduced and two groups merged (**b**). PC-LDA shows a clear reduction in class overlap in OMB (using 50 PCs)(i), which can also be observed in the kernel density (ii) and scatter plots (iii) produced in R (using 55 PCs). Random forest analysis, using a 70%/30% ratio for training and testing, resulted in identification accuracies of 93% for 1-day-old specimens and 89% for 3–4 day old mosquitoes (iv). Sample numbers per class for the model in **a**: 1 day (71), 2 days (28), 3 days (21), 4 days (31). Sample numbers per class for the model in **b**:1 day (55), 3–4 days (52) — sample numbers from the 1-day class were reduced for random forest analysis

Larvae of *Ae. detritus* collected in the Neston area were allowed to develop to adults and were harvested at different ages. We used these adults to establish whether age-specific patterns could be retained in the presence of confounding factors, such as impact of the natural environment during larval development. PC-LDA of 0–4-day-old *Ae. detritus* mosquitoes (males and females) resolved into four age classes (1 day, 2 days, 3 days and 4 days) with clear resolution of the four groups (Fig. 8a). As expected for continous data, the classes are very close or slightly overlap, consistent with the previous analysis of 0–5 day old *An. gambiae (*Fig. 5). The three age groups ranging from 2 to 4 days were resolved by LD 2 and 3, whereas mosquitoes that had just emerged are separated by the largest variance component (LD 1, Fig. 8a). This finding is particularly encouraging, as it matches previous age models based on laboratory-raised specimens. To test more separated classes, in a subsequent analysis, the 2-day class was removed and additionally, the two older age groups were combined to increase separation and sample numbers. Separation of the two new age classes was clearly improved when analysed through PC-LDA in Offline Model Builder, repeated PC-LDA in R replicated the result, showing only a few samples are being misclassified in the process (Fig. 8b). The data matrix exported from OMB was also subjected to random forest analysis, based on 70% training data/30% evaluation data. The 1-day-old mosquitoes reached a correct identification rate of 93%, of the 3–4-day mosquitoes 89% were correctly identified (Fig. 8b). Aggregated age classes, with at least a 24-h period between them, are clearly beneficial for class separation and the gain in performance more than offsets the loss of resolution.

**Fig. 9:**
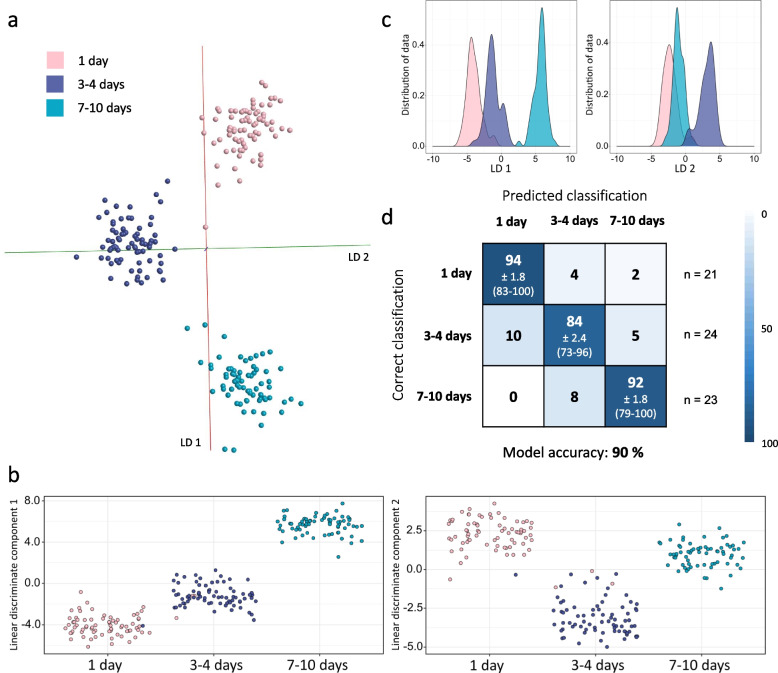
Species independent separation of age groups using wild-derived mosquitoes. In addition to the *Ae. detritus* samples used in Fig. 8, samples from four other species and another age class (7–10 days) were introduced, including adult mosquitoes that were kept with or without water or fed sucrose solution. A portion of specimens was killed by freezing, some died due to other reasons shortly before being collected; all were stored at − 20 °C before analysis. PC-LDA achieved separation of the three age groups (1 day, 3–4 days, 7–10 days), as can be seen in the OMB model (based on 100 PCs) (**a**) as well as the scatterplots (**b**) and kernel density distributions (**c**) created in R (based on 95 PCs). An overall 90% of test samples were correctly identified during random forest analysis with group-specific accuracies of 94% for newly emerged specimens (1 day old), 84% for 3–4-day-old mosquitoes and 92% for the 7–10-day-old group (**d**). Sample numbers per class: 1 day (75), 3–4 days (75), 7–10 days (69)

The laboratory mosquito-based age model proved to be stable even in the presence of multiple species. To test whether this also holds true for wild-derived specimens, mosquitoes from four different species (*Aedes detritus*, *Culiseta annulata*, *Aedes rusticus*, *Aedes punctor)* were used to build age models with the same classifications as seen in Fig. 8 (Additional file 2: Sup. Figure 22). The average percentage of samples correctly identified through random forest dropped only slightly from 91% (Fig. 9b) to 88% upon the introduction of different species. As before, this analysis was performed with combined classes and a 1-day gap between them. Reduction of classes alone can increase separation performance significantly, which was tested using 1-day and 3–4-day classes. A comparison of PC-LDA models, built using different sets of classes, shows the step-wise improvement of separation for the single-species as well as the multi-species model (Additional files 2: Sup. Figures 23 and 25). All models were cross-validated in Offline Model Builder (Additional file 2: Sup. Figures 24 and 26).

After successful separation of age groups using wild-derived mosquitoes, which were unfed and killed by freezing, more samples were added to the existing 0–1 day/3–4 days model to test the stability of pattern-based age grading (Fig. 9). A broader range of samples was included in this extended model, purposely adding variance and potentially confounding factors. A third age group was introduced to represent older mosquitoes, ranging from 7 to 10 days. Whilst no intervention took place between emergence and killing when raising the previously used wild-derived mosquitoes (Figs. 3 and 8), these adults were raised in three different conditions: kept dry, kept with water or fed with sucrose solution. Furthermore, samples from different species were added; four species (*Ae. detritus*,* Ae. rusticus*,* Ae. punctor *and* Cs. annulata*) are represented in the first two age classes (1 day and 3–4 days) and 2 species (*Ae. detritus *and* Ae. caspius*) are part of the older age class (168–240 h). Whereas all 1-day-old specimens had been killed by freezing, some mosquitoes of the older age classes had died naturally before collection. They were nonetheless added to the sample pool to add further variance to the model. Linear discriminant analysis in OMB, based on 100 principal components, produced a distinct separation of the three age groups (Fig. 9a). Similar results can be seen when conducting LD analysis (based on 95 PCs) in R, the scatterplots show that only a few samples are confused between the 1-day and 3–4-day classes and that all 7–10 day old mosquitoes are correctly located (Fig. 9b). The latter can be explained when viewing the variance distribution in the kernel density plots; the oldest group is separated clearly via LD 1, whereas separation of 1 day and 2–3 day is mostly based on LD 2, meaning there is a slightly greater variance benefitting the distinction of 7–10-day-old specimens (Fig. 9c). In random forest analysis, the 1-day-old test samples scored the highest identification accuracy with 94%, followed by the 7–10-day-old class with 92% of samples currently identified and the 3–4-day-old group with 84% correct identification (Fig. 9d).

All three age models for wild-derived specimens were re-built in OMB after randomly assigning them age classifications to ensure that previous separations had been achieved through age-related variance (Additional file 2: Sup. Figure 27). A comparison of cross-validation results of models with correct or randomly assigned classifications underpins the presence of age-related differences in the collected data; classification rates for models with random sample assignment are drastically lower (Additional file 2: Sup. Figure 28). Even reducing the number of principal components and therefore variance used for LD analysis did not hinder class separation, it merely reduced the extent of separation and class resolution (Additional file 2: Sup. Figure 29).

The ion bins used to age-grade the semi-wild mosquitoes during random forest analysis differ from the bins identified for the age model based on laboratory-raised specimens (Additional file 2: Sup. Figure 30). This could be caused by environmental effects during their immature stages, i.e. the difference between laboratory and field pools, but could also be related to other factors, such as different raising conditions during adulthood, differing storage conditions (room temperature and freezer) or perhaps the ageing pattern simply varies for different mosquito species. Whilst these experiments prove that age-grading through REIMS can be extended beyond mosquitoes raised in controlled laboratory environments, it is yet unknown whether models based on laboratory-raised or semi-wild mosquitoes (derived from the wild as immatures) are capable of age-grading fully-wild mosquitoes, which will require further exploration.

## Discussion

A mass spectrometric method such as REIMS has compelling advantages in the context of field studies. First, because there is minimal sample preparation, the generation of the aerosol can be applied to a broad range of biological material, including tissues, [32–34], foodstuffs [35–37, 52], faecal pellets [42], bacterial colonies [38–40] and plant materials [53]. Within a few seconds, the burn event generates a complex, richly detailed mass spectrum. Atypically, there is no attempt to interpret this spectrum in terms of the molecular species that are introduced into the instrument. Rather, it is the pattern of ions that create a complex vector, and the matrix of samples and *m/z* bins are suitable for multivariate data analysis whether supervised or unsupervised.

We have primarily explored the ability of REIMS to serve as a rapid method to create species and age profiles of mosquitoes using supervised machine learning approaches such as PC-LDA and random forest. This information on mosquito demography is key for implementing and assessing the impact of vector control strategies, as even a small change in the lifespan of mosquito species that are vectors of disease can have a big impact on pathogen transmission. Models generated using REIMS data can resolve the three main vectors of malaria in the *An. gambiae* species complex. Furthermore, seven species belonging to the Culicidae family were distinguished with an accuracy of over 90%. This identification accuracy is comparable to that reported by the MIRS method coupled with machine learning for lab-raised *An. gambiae* and *An. arabiensis* [26] and the recently developed deep-learning MIRS models [54].

REIMS could also discriminate three age groups of lab-raised *Anopheles* mosquitoes and three age groups of wild-derived *Culicine* mosquitoes with an accuracy of over 90%. By comparison, NIRS can place lab-reared *An. gambiae s.s* into binary groups (less than and greater than 7 days old) with an accuracy of 80% [29] up to day 7 [30]. Likewise, MIRS prediction of chronological age is highly variable with the very young (1 day) and very old (15 days) mosquitoes being more accurately predicted than intermediate ages. A deep-learning MIRS model achieved an accuracy of 89% for the resolution of wild-derived *Anopheles* mosquitoes into three age classes (1–4, 5–10 and 11–17 days old) [28]. Thus, REIMS delivers a species and age-predictive accuracy that is comparable to other analytical methods. This performance was maintained with field-derived samples and is robust to field collection and storage, critical steps for translational development. Ultimately, the resolution and granularity of age classification, converting a continuous variable into categories, will be dictated by field conditions and the use to which such information is put.

There are several other factors that need to be addressed for an age-grading methodology to become a valuable field tool. First, the methodology must be compatible with samples collected under field conditions — the ability of REIMS to resolve desiccated rather than frozen samples is advantageous.

Secondly, age predictions must not be unduly influenced by temperature and humidity, diet, intraspecific phenotypic variation or physiological status (blood fed, gravid etc.). Whilst results from *Culicines* sampled from the UK are encouraging, further studies of the robustness of the predictions under field conditions are needed.

Thirdly, although the mass spectrometry data does not require interpretation or molecular assignment, in the current configuration, the REIMS source is coupled to a relatively sophisticated mass spectrometer; to reduce the capital expenditure it would be desirable to explore instrumentation providing less resolution. Currently, data is binned in 0.1 m*/z* increments to reduce complexity prior to multivariate analysis. If extended to 1 m*/z* wide bins, the resulting data matrix can still be used to separate species as well as age classes (Additional file 2: Sup. Figure 31). This opens the possibility of coupling the REIMS source to a lower-resolution instrument. Alternatively, a more focused analysis could be created that emphasises specific ions, for example by isolation of precursor ions, fragmentation and quantification of product ions (selected reaction monitoring). Both approaches would improve compatibility with more routine instrumentation, such as single or triple quadrupole instruments, that would have a substantially lower cost.

Fourthly, at present, the robustness of the models that are created is not fully resolved. Apart from issues such as instrument drift and source variation, the extent to which time-dependent or geography-dependent differences in subjects might influence responsiveness to a single model is unclear. It is possible that ‘local’ models (in time or space) might improve separation.

Finally, although acquisition and recognition are extremely rapid, there is scope for the evolution of multivariate data methods that might increase confidence and the speed of profiling. The combination of a simple, rapid ionisation method with real-time data acquisition and data reduction makes REIMS worthy of further consideration. Recent progress in the ability of other methods to determine species and age, notably the DL-MIRs approach and the qPCR method to age spermatozoa stored in females, is also encouraging and further studies using multiple approaches on the same sample sets will be important to determine the robustness of each method; this should include a comparison of the fixed and variable costs, and ease of data interpretation.

## Conclusions

Rapid evaporative ionisation mass spectrometry is an effective tool for the generation of informative mass spectra from an intact insect specimen, with no prior sample preparation. The spectra are rich in detail, predominantly representing the lipid content of the body. The raw spectra can be binned and analysed using machine learning approaches to yield information on species and age of the subject, without interpretation of the spectra. The performance of REIMS is comparable to other methodologies, with the potential for higher throughput analysis, and this analytical method may drive or support other methods, and generate orthogonal data sets. Such multivariate approaches have considerable potential for the profiling of wild mosquitoes, particularly where they are key vectors of disease.

## Methods

### Laboratory-raised mosquitoes

Three *Anopheles* mosquito strains maintained at the Liverpool School of Tropical Medicine were used in this study: an *An. gambiae s.s* strain called Kisumu (Kenya, 1975), an *An. coluzzii* strain called Ngousso (Cameroon, 2006) and an *An. arabiensis* strain called Moz (Mozambique, 2009). All three strains were reared at 26 ± 2 °C and a relative humidity (RH) of 80 ± 10% under a L12:D12 h light:dark cycle with a 1-h dawn and dusk. All stages of larvae were reared in distilled water and fed on ground fish food (Tetramin tropical flakes, Tetra, Blacksburg, VA, USA). Adults were provided with 10% sucrose solution ad libitum.

*Anopheles* laboratory-raised mosquitoes were collected as pupae and placed in paper buckets for a 24-h emergence period (day 0). Thereafter non emerged pupae were removed or kept for an additional 24 h (in cases where two consecutive age groups are reported). Age profiling samples were collected at different days post emergence, as depicted in the results section. Females were separated from males based on clear morphological differences (sexual dimorphism of the antenna) and aspirated into paper buckets; males were discarded. Mosquitoes were killed either by freezing at − 20 °C, in which case samples were stored in the freezer until the day of analysis, or through dehydration by placing the buckets at 36–38 °C overnight without a water source. In the latter case, samples were stored the next day in plastic tubes with silica gel at room temperature until the day of analysis.

### Wild-caught mosquitoes

*Ae. detritus* and *Ae. caspius* larvae were collected from pools in the Dee estuary, as outlined in [46]. Larvae were collected from their natural breeding pools and raised to adults in the same water as they were derived. Adult mosquitoes were raised throughout the year, killed by freezing and stored at − 20 °C for different lengths of time before being analysed by REIMS on different days spread over the course of several months. All specimens were analysed in randomised order. Other species were collected as larvae from pools at locations in the Neston area (summarised in Additional file 2: Sup. Figure 32). Specimens that were analysed in their larval stage were filtered and rinsed with MilliQ water 2–3 times before being killed by freezing. Specimens that were collected as larvae and then analysed in their adult stage were reared in glass jars in their original water and fed intermittently with yeast (Zipvit Brewer’s yeast). After emerging as adults, they were captured in nets attached to the original container that was emptied every 24 h. Adults were killed by freezing, and their species and sex were identified morphologically according to standard keys [47, 48]. The pool of larval origin, date of larval collection, date of emergence and date of killing (and thus, adult age at death), sex and species were recorded for each individual. All samples were then stored at − 20 °C until analysis.

For larval analysis, we compared larvae from readily identified pools that are essentially monocultures of a single mosquito species. This is based on data from 2018 to 2022. Over this period, from the pool that produces *Aedes detritus*, 248 adults that emerged from 248 larvae have been *Aedes detritus*. This pool is a small (2 × 1 m) hollow in grass with mud at the bottom, brackish from being inundated with seawater several times a year. For the pool that produces *Aedes punctor*, the comparable statistic is 379 out of 380. This is a larger pool (5 × 4 m) in a pine wood, filled with rainwater from autumn until early summer, and has a rather acidic soil/organic debris base. Both pools are classical for each species.

### REIMS analysis

Samples were analysed via a rapid evaporative source (REIMS, Waters, Wilmslow, UK) attached to a Synapt G2Si instrument ion mobility equipped quadrupole time of flight mass spectrometer (Waters, UK). The specimens were burned/evaporated using a monopolar electrosurgical pencil (Erbe Medical UK Ltd, Leeds, UK), which was connected to a VIO 50 C electrosurgical generator (Erbe Medical UK Ltd, Leeds, UK), providing electrical current, and to the source inlet via plastic tubing. A black conductive rubber mat, placed underneath the samples, acted as a counter electrode and facilitated the flow of electric current. To avoid inhalation of fumes during analysis, the burning process was performed within a fume box (Air Science, Lydiate, Merseyside, UK). Insects were analysed using a 40-W setting on the generator and the cutting option of the pencil. To increase conductivity and to protect the counter electrode during analysis, specimens were placed on a piece of glass microfibre paper (GFP, GE Healthcare Whatman) on top of a wet paper surface (moistened with MilliQ water).

Whilst burning the entire biomass of single specimens, the aerosol was aspirated through the pencil and the attached 3 m long tubing into the REIMS source, using a nitrogen-powered venturi valve on the source inlet. To increase the aerosol capture, a wide-bore piece of plastic tubing was additionally placed over the tip of the electrosurgical pencil. A whistle incorporated into the venturi tube guided the aerosol as well as a lock mass solution of leucine enkephalin (Waters, UK) in propan-2-ol (CHROMASOLV, Honeywell Riedel-de-Haën) into the source. This also filters the incoming aerosol to prevent larger particles from entering the inlet capillary. Inside the source, the ionised particles were declustered through contact with a heated impactor (Kanthal metal coil at 900 °C).

Mass spectra were acquired in negative ion mode at a rate of 1 scan per second between 50 m*/z* and 1200 m*/z*. The sample cone and heater bias were set to 60 V. Instrument calibration was performed daily in resolution mode using a 0.5-mM solution of sodium formate (flow rate 50 µl/min). The lock mass solution (0.4 µg/ml, leucine enkephalin in IPA) was continuously introduced during sample analysis at 30 µl/min. For each experiment, samples were analysed in a formally randomised order on 1 or more days.

### Data analysis

The raw mass spectra were imported into the model building software package Offline Model Builder (OMB-1.1.28; Waters Research Centre, Hungary), which allows separation of sample groups (classifications) based on principal component analysis (PCA) and linear discriminant analysis (LDA). Data were additionally analysed using R (version 3.6.1) [55] and the R Studio environment [56], by PCA and LDA, as well as random forest analysis.

For Offline Model Builder, the burn events of the analysed specimens were defined individually, summing all the MS scans within each chosen area. The option to create one spectrum per sample was selected. Other pre-processing parameters included the intensity threshold, which was set between 4e5 and 9e5 (depending on the background baseline), spectra correction using the lock mass (leucine enkephalin, 554.26 m*/z*) and background subtraction. To reduce the complexity of the mass spectral data, all acquired data points from 50 to 1200 m*/z* were combined into mass bins, each 0.1 m*/z* wide. Subsequent model calculation was based on principal component and linear discriminant analysis (PCA-LDA).

The models built by Offline Model Builder were cross-validated (leaving out 20% of data, for outliers the standard deviation multiplier was set to 5) to obtain the correct classification rate, as well as the number of failures and outliers and a matrix displaying the number of correctly and incorrectly identified samples of each classification. To additionally test discrimination, sample classifications were randomised and the data were re-analysed, the expectation being a random distribution of samples and the inability to achieve separation.

For further analysis with R, the data matrix of each model was exported as a. csv file from Offline Model Builder, containing information about classification and the relative intensities for every mass bin. The matrices were used to perform random forest analysis in R using the package ‘randomForest’ [57]. The data sets were randomly split into a training set (approx. 70% of the data) and a test set (approx. 30% of the data). Random forest results are displayed in form of confusion matrices. Trees were conducted 10 times for every model (using a different, randomly selected subset of samples for training and testing every time); the numbers of correctly identified and confused samples were turned into percentages and averaged. The optimal number of trees and *mtry* value were determined during the first analysis of each model and kept the same for each repeated analysis. A second R package, called ‘randomForestExplainer’ [58], was used to identify the most informative bins/ions that were driving class separation. PCA-LDA was also performed within the R environment, using the in-built package ‘stats’ and the package ‘MASS’ [59] and results visualised in form of kernel density plots and 2D- and 3D-scatter plots created using ‘ggplot2’ [60] and ‘scatterplot3d’ [61]. PC-LDA-based models were built with the number of principal components (PCs) giving the best separation before overfitting the model (that number can slightly vary between OMB and R). To ensure that separation of classes is still possible when using less variance, models were also built using only a quarter of the maximum number of PCs possible for each model.

## Supplementary Information

**Additional file 1: Video file 1.** The REIMS ionization process.

**Additional file 2: Supplementary Figures 1-31.** Additional data analyzes and randomisation confirmatory tests. **Sup. Fig. 1.** REIMS spectra from three *Anopheles* species; **Sup. Fig. 2.** Randomisation analysis of *Anopheles* species; **Sup. Fig. 3.** Effect of number of principal components on separation of *Anopheles* species; **Sup. Fig. 4.** Cross-validation results of Anopheles species models built with correct and randomly assigned classifications; **Sup. Fig. 5.** Cross-validation and random forest results of species separation of UK mosquitoes ; **Sup. Fig. 6.** Randomisation analysis of seven species data set using UK mosquitoes; **Sup. Fig. 7.** Species identification results for different sample cohorts; **Sup. Fig. 8.** Species identification at the larval stage; **Sup. Fig. 9.** Age resolution of *Anopheles* mosquitoes with fewer principal components; **Sup. Fig. 10.** Randomisation analysis of age separation of *Anopheles* mosquitoes; **Sup. Fig. 11.** Age variation in REIMS spectra; **Sup. Fig. 12.** Reduction in age classes improves separation; **Sup. Fig. 13.** Cross validation of age determination by REIMS; **Sup. Fig. 14.** Cross validation of Anopheles species and age models; **Sup. Fig. 15.** Age separation with fewer principal components; **Sup. Fig. 16.** Ion bins dominating separation of three Anopheles species and three age groups; **Sup. Fig. 17.** Cross validation of two factor model; **Sup. Fig. 18.** Random forest classification of two factor model; **Sup. Fig. 19.** PC-LDA analysis of two factor model; **Sup. Fig. 20.** Ion bins dominating separation in two factor model; **Sup. Fig. 21.** Randomisation test of Anopheles species, age and two factor models; **Sup. Fig. 22.** Age separation of wild derived mosquito populations; **Sup. Fig. 23.** Age separation model of *Aedes detritus; ***Sup. Fig. 24.** Cross validation of *Ae. detritus* age model; **Sup. Fig. 25.** Age separation model for four wild-derived mosquito species; **Sup. Fig. 26.** Cross validation of multi-species age model; **Sup. Fig. 27.** Randomisation test of age determination models of wild-derived mosquitoes; **Sup. Fig. 28.** Cross-validation results of the different age models with correctly and randomly assigned classes; **Sup. Fig. 29.** Linear discriminant distributions for the three age models; **Sup. Fig. 30.** Ion bins dominating separation in the age model with wild-derived mosquitoes; **Sup. Fig. 31.** Anopheles age and species models built with larger m/z bins; **Sup. Fig. 32.** Coordinates of the locations where immature mosquito specimens were collected.

## Data Availability

All raw data files are freely available in the University of Liverpool data repository: Beynon, Robert, Wagner, Iris and Ranson, Hilary (2021) Rapid identification of mosquito species, sex and age by mass spectrometric analysis. RAW MS data files. [Data Collection] (http://dx.doi.org/10.17638/datacat.liverpool.ac.uk/1565) [62]. These comprise 2920 Waters MassLynx raw files.

## Abbreviations

REIMS: Rapid evaporative ionisation mass spectrometry
EIP: Extrinsic incubation period
MALDI-TOF: Matrix-assisted laser desorption ionisation time of flight mass spectrometry
LD: Linear discriminant
MIRS: Mid-range infrared spectroscopy
*m/z*: Mass-to-charge ratio
NIRS: Near-infrared spectroscopy
OMB: Offline model builder
PC-LDA: Principal component linear discriminant analysis
PCA: Principal component analysis
PC: Principal component

## References

[1] WHO (2021). World Malaria Report.

[2] Wilder-Smith A, Gubler DJ, Weaver SC, Monath TP, Heymann DL, Scott TW (2017). Epidemic arboviral diseases: priorities for research and public health. Lancet Infect Dis.

[3] Bhatt S, Weiss DJ, Mappin B, Dalrymple U, Cameron E, Bisanzio D, Smith DL, Moyes CL, Tatem AJ, Lynch M, Fergus CA, Yukich J, Bennett A, Eisele TP, Kolaczinski J, Cibulskis RE, Hay SI, Gething PW (2015). Coverage and system efficiencies of insecticide-treated nets in Africa from 2000 to 2017. Elife.

[4] Ranson H, Lissenden N (2016). Insecticide resistance in african anopheles mosquitoes: a worsening situation that needs urgent action to maintain malaria control. Trends Parasitol.

[5] Dusfour I, Vontas J, David J-P, Weetman D, Fonseca DM, Corbel V, Raghavendra K, Coulibaly MB, Martins AJ, Kasai S (2019). Management of insecticide resistance in the major Aedes vectors of arboviruses: advances and challenges. PLoS Negl Trop Dis.

[6] Achee NL, Grieco JP, Vatandoost H, Seixas G, Pinto J, Ching-Ng L, Martins AJ, Juntarajumnong W, Corbel V, Gouagna C, David JP, Logan JG, Orsborne J, Marois E, Devine GJ, Vontas J (2019). Alternative strategies for mosquito-borne arbovirus control. PLoS Negl Trop Dis.

[7] Quinn CM, Nolan T (2020). Nuclease-based gene drives, an innovative tool for insect vector control: advantages and challenges of the technology. Curr Opin Insect Sci.

[8] Beier JC (1998). Malaria parasite development in mosquitoes. Annu Rev Entomol.

[9] Chan M, Johansson MA (2012). The incubation periods of Dengue viruses. PLoS One.

[10] Musso D, Gubler DJ (2016). Zika Virus. Clin Microbiol Rev.

[11] Gillies MT, Wilkes TJ (1965). A study of the age-composition of populations of Anopheles gambiae Giles and A. funestus Giles in North-Eastern Tanzania. Bull Entomol Res.

[12] Kamiya T, Greischar MA, Wadhawan K, Gilbert B, Paaijmans K, Mideo N (2019). Temperature-dependent variation in the extrinsic incubation period elevates the risk of vector-borne disease emergence. Epidemics.

[13] Muir LE, Kay BH (1998). Aedes aegypti survival and dispersal estimated by mark-release-recapture in northern Australia. Am J Trop Med Hyg.

[14] Desena ML, Edman JD, Clark JM, Symington SB, Scott TW (1999). Aedes aegypti (Diptera: Culicidae) age determination by cuticular hydrocarbon analysis of female legs. J Med Entomol.

[15] Cook PE, Hugo LE, Iturbe-Ormaetxe I, Williams CR, Chenoweth SF, Ritchie SA, Ryan PA, Kay BH, Blows MW, O’Neill SL (2006). The use of transcriptional profiles to predict adult mosquito age under field conditions. Proc Natl Acad Sci U S A.

[16] Hugo LE, Kay BH, O’Neill SL, Ryan PA (2010). Investigation of environmental influences on a transcriptional assay for the prediction of age of Aedes aegypti (Diptera: Culicidae) mosquitoes. J Med Entomol.

[17] Joy TK, Jeffrey Gutierrez EH, Ernst K, Walker KR, Carriere Y, Torabi M, Riehle MA (2012). Aging field collected Aedes aegypti to determine their capacity for dengue transmission in the southwestern United States. PLoS One.

[18] Hugo LE, Monkman J, Dave KA, Wockner LF, Birrell GW, Norris EL, Kienzle VJ, Sikulu MT, Ryan PA, Gorman JJ, Kay BH (2013). Proteomic biomarkers for ageing the mosquito Aedes aegypti to determine risk of pathogen transmission. PLoS One.

[19] Iovinella I, Caputo B, Michelucci E, Danidella Torre FRA (2015). Candidate biomarkers for mosquito age-grading identified by label-free quantitative analysis of protein expression in Aedes albopictus females. J Proteomics.

[20] Sikulu MT, Monkman J, Dave KA, Hastie ML, Dale PE, Kitching RL, Killeen GF, Kay BH, Gorman JJ, Hugo LE (2015). Proteomic changes occurring in the malaria mosquitoes Anopheles gambiae and Anopheles stephensi during aging. J Proteomics.

[21] Nabet C, Chaline A, Franetich JF, Brossas JY, Shahmirian N, Silvie O, Tannier X, Piarroux R (2020). Prediction of malaria transmission drivers in Anopheles mosquitoes using artificial intelligence coupled to MALDI-TOF mass spectrometry. Sci Rep.

[22] Yssouf A, Socolovschi C, Flaudrops C, Ndiath MO, Sougoufara S, Dehecq JS, Lacour G, Berenger JM, Sokhna CS, Raoult D, Parola P (2013). Matrix-assisted laser desorption ionization–time of flight mass spectrometry: an emerging tool for the rapid identification of mosquito vectors. PLoS One.

[23] Yssouf A, Parola P, Lindström A, Lilja T, L’Ambert G, Bondesson U, Berenger JM, Raoult D, Almeras L (2014). Identification of European mosquito species by MALDI-TOF MS. Parasitol Res.

[24] Johnson BJ, Hugo LE, Churcher TS, Ong OTW, Devine GJ (2020). Mosquito age grading and vector-control programmes. Trends Parasitol.

[25] Madan D, Rivera R, Ortega C, Touchon JC, Kimball C, van Gemert GJ, Graumans W, Matsuura S, Parghi SS, Bell D, Bousema T, Drakeley C, Collins KA, Burkot TR (2022). Estimating female malaria mosquito age by quantifying Y-linked genes in stored male spermatozoa. Sci Rep.

[26] González Jiménez M, Babayan SA, Khazaeli P, Doyle M, Walton F, Reedy E, Glew T, Viana M, Ranford-Cartwright L, Niang A, Siria DJ, Okumu FO, Diabaté A, Ferguson HM, Baldini F, Wynne K (2019). Prediction of mosquito species and population age structure using mid-infrared spectroscopy and supervised machine learning. Wellcome Open Res.

[27] Lambert B, Sikulu-Lord MT, Mayagaya VS, Devine G, Dowell F, Churcher TS (2018). Monitoring the age of mosquito populations using near-infrared spectroscopy. Sci Rep.

[28] Siria DJ, Sanou R, Mitton J, Mwanga EP, Niang A, Sare I, Johnson PCD, Foster GM, Belem AMG, Wynne K, Murray-Smith R, Ferguson HM, González-Jiménez M, Babayan SA, Diabaté A, Okumu FO, Baldini F (2022). Rapid age-grading and species identification of natural mosquitoes for malaria surveillance. Nat Commun.

[29] Mayagaya VS, Michel K, Benedict MQ, Killeen GF, Wirtz RA, Ferguson HM, Dowell FE (2009). Non-destructive determination of age and species of Anopheles gambiae s.l. using near-infrared spectroscopy. Am J Trop Med Hyg.

[30] Krajacich BJ, Meyers JI, Alout H, Dabiré RK, Dowell FE, Foy BD (2017). Analysis of near infrared spectra for age-grading of wild populations of Anopheles gambiae. Parasit Vectors.

[31] Lyimo IN, Ferguson HM (2009). Ecological and evolutionary determinants of host species choice in mosquito vectors. Trends Parasitol.

[32] Balog J, Sasi-Szabó L, Kinross J, Lewis MR, Muirhead LJ, Veselkov K, Mirnezami R, Dezső B, Damjanovich L, Darzi A, Nicholson JK, Takáts Z (2013). Intraoperative tissue identification using rapid evaporative ionization mass spectrometry. Sci Transl Med.

[33] Hänel L, Kwiatkowski M, Heikaus L, Schlüter H (2019). Mass spectrometry-based intraoperative tumor diagnostics. Future Sci OA.

[34] Santilli AML, Jamzad A, Janssen NNY, Kaufmann M, Connolly L, Vanderbeck K, Wang A, McKay D, Rudan JF, Fichtinger G, Mousavi P (2020). Perioperative margin detection in basal cell carcinoma using a deep learning framework: a feasibility study. Int J Comput Assist Radiol Surg.

[35] Balog J, Perenyi D, Guallar-Hoyas C, Egri A, Pringle SD, Stead S, Chevallier OP, Elliott CT, Takats Z (2016). Identification of the species of origin for meat products by rapid evaporative ionization mass spectrometry. J Agric Food Chem.

[36] Black C, Chevallier OP, Haughey SA, Balog J, Stead S, Pringle SD, Riina MV, Martucci F, Acutis PL, Morris M, Nikolopoulos DS, Takats Z, Elliott CT (2017). A real time metabolomic profiling approach to detecting fish fraud using rapid evaporative ionisation mass spectrometry. Metabolomics.

[37] Ross A, Brunius C, Chevallier O, Dervilly G, Elliott C, Guitton Y, et al. Making complex measurements of meat composition fast: application of rapid evaporative ionisation mass spectrometry to measuring meat quality and fraud. Meat Sci. 2021;181:10833. 10.1016/j.meatsci.2020.108333 33067082

[38] Cameron SJ, Bolt F, Perdones-Montero A, Rickards T, Hardiman K, Abdolrasouli A, Burke A, Bodai Z, Karancsi T, Simon D, Schaffer R, Rebec M, Balog J, Takáts Z (2016). Rapid Evaporative Ionisation Mass Spectrometry (REIMS) provides accurate direct from culture species identification within the genus candida. Sci Rep.

[39] Strittmatter N, Jones EA, Veselkov KA, Rebec M, Bundy JG, Takats Z (2013). Analysis of intact bacteria using rapid evaporative ionisation mass spectrometry. Chem Commun (Camb).

[40] Strittmatter N, Rebec M, Jones EA, Golf O, Abdolrasouli A, Balog J, Behrends V, Veselkov KA, Takats Z (2014). Characterization and identification of clinically relevant microorganisms using rapid evaporative ionization mass spectrometry. Anal Chem.

[41] Sarsby J, McLean L, Harman VM, Beynon RJ. Monitoring recombinant protein expression in bacteria by rapid evaporative ionisation mass spectrometry. Rapid Commun Mass Spectrom. 2019;35(supp 2):e8670. 10.1002/rcm.8670 PMC8047878 31760669

[42] Davidson NB, Koch NI, Sarsby J, Jones E, Hurst JL, Beynon RJ (2019). Rapid identification of species, sex and maturity by mass spectrometric analysis of animal faeces. BMC Biol.

[43] Wagner I, Koch NI, Sarsby J, White N, Price TAR, Jones S, Hurst JL, Beynon RJ (2020). The application of rapid evaporative ionization mass spectrometry in the analysis of Drosophila species-a potential new tool in entomology. Open Biol.

[44] Koella JC, Lyimo EO (1996). Variability in the relationship between weight and wing length of Anopheles gambiae (Diptera: Culicidae). J Med Entomol.

[45] Fontaine MC, Pease JB, Steele A, Waterhouse RM, Neafsey DE, Sharakhov IV, Jiang X, Hall AB, Catteruccia F, Kakani E, Mitchell SN, Wu YC, Smith HA, Love RR, Lawniczak MK, Slotman MA, Emrich SJ, Hahn MW, Besansky NJ (2015). Mosquito genomics. Extensive introgression in a malaria vector species complex revealed by phylogenomics. Science.

[46] Clarkson MJ, Enevoldson TP (2021). The factors which influence the breeding and number of Aedes detritus in the Neston area of Cheshire, UK, the production of a local mosquito forecast and public bite reporting. J Eur Mosq Control Assoc.

[47] Cranston P, Ramsdale C, Snow K, White GB. Adults, larvae and pupae of British mosquitoes (Culicidae): a key. Freshwater Biological Association Scientific Publications No. 48. Ambleside, UK: Freshwater Biological Association; 1987.

[48] Snow KR (2015). Naturalists’ Handbooks.

[49] Harbach RE (2007). The Culicidae (Diptera): a review of taxonomy, classification and phylogeny. Zootaxa.

[50] Vontas J, Moore S, Kleinschmidt I, Ranson H, Lindsay S, Lengeler C, Hamon N, McLean T, Hemingway J (2014). Framework for rapid assessment and adoption of new vector control tools. Trends Parasitol.

[51] Ryan SJ, Ben-Horin T, Johnson LR (2015). Malaria control and senescence: the importance of accounting for the pace and shape of aging in wild mosquitoes. Ecosphere.

[52] Barlow RS, Fitzgerald AG, Hughes JM, McMillan KE, Moore SC, Sikes AL, Tobin AB, Watkins PJ (2021). Rapid evaporative ionization mass spectrometry: a review on its application to the red meat industry with an Australian context. Metabolites.

[53] Arena K, Rigano F, Mangraviti D, Cacciola F, Occhiuto F, Dugo L, Dugo P, Mondello L (2020). Exploration of rapid evaporative-ionization mass spectrometry as a shotgun approach for the comprehensive characterization of Kigelia Africana (Lam) Benth. Fruit. Molecules.

[54] Siria DJ, Sanou R, Mitton J, Mwanga EP, Niang A, Sare I, et al. Rapid ageing and species identification of natural mosquitoes for malaria surveillance. Nat Commun. 2022;13:1501. 10.1038/s41467-022-28980-8 PMC8938457 35314683

[55] Team RC (2020). R: A language and environment for statistical computing.

[56] Team RS (2020). RStudio: Integrated Development for R.

[57] Liaw A, Wiener M (2002). Classification and regression by randomForest. R news.

[58] Paluszynska A, Biecek P, Jiang Y (2017). Package ‘randomForestExplainer’. Explaining and visualizing random forests in terms of variable importance.

[59] Venables WN, Ripley BD. Modern applied statistics with S. New York: Springer; 2002.

[60] Wickham H. ggplot2:Elegant Graphics for Data Analysis. New York: Springer-Verlag; 2016.

[61] Ligges U, Machler M (2003). Scatterplot3d - an R Package for Visualizing Multivariate Data. J Stat Softw.

[62] Beynon R, Wagner I, Ranson H. Rapid identification of mosquito species, sex and age by mass spectrometric analysis. RAW MS data files. [Data Collection]; 2021. 10.17638/datacat.liverpool.ac.uk/1565. 10.1186/s12915-022-01508-8 PMC9872345 36690979

